# In silico data analyses of the hotspot mutations of *CHM* gene in choroideremia disease

**DOI:** 10.1016/j.dib.2018.04.023

**Published:** 2018-04-12

**Authors:** Saber Imani, Iqra Ijaz, Marzieh Dehghan Shasaltaneh, Shangyi Fu, Jingliang Cheng, Junjiang Fu

**Affiliations:** a Key Laboratory of Epigenetics and Oncology, Research Center for Preclinical Medicine, Southwest Medical University, Luzhou, Sichuan, China; b Chemical Injuries Research Center, Baqiyatallah Medical Sciences University (BMSU), Tehran, Iran; c Laboratory of Neuro-organic Chemistry, Institute of Biochemistry and Biophysics (IBB), University of Tehran, Tehran, Iran; d Laboratory of Systems Biology and Bioinformatics (LBB), Institute of Biochemistry and Biophysics, University of Tehran, Tehran, Iran; e The Honors College, University of Houston, Houston, TX, USA; f Department of Molecular and Human Genetics, Baylor College of Medicine, Houston, Texas 77030, USA; g Hunan Normal University Medical College, Changsha, Hunan, China

**Keywords:** In silico, Choroideremia, Rab escort protein 1, Molecular dynamic simulation

## Abstract

This data article provides compelling computational analysis of the hotspot *CHM* gene mutations that contribute to the progressive causativeness and susceptibility of Choroideremia in patients. We performed structural and molecular dynamics (MD) simulation analysis on abnormal states of the CHM protein caused by deleterious and disease-causing hotspot mutant forms of CHM: S89C, E177K, and V529H. Within 40 ns, MD simulation time composed of the E177K mutant shows conformational alteration especially in several parts of the variant. Mathematically, we applied eigenvector analysis to determine the modes of flexibility and atomic positional fluctuations that contribute significantly to the overall motion of the CHM protein in terms of structural alteration, free energy landscapes (FEL), entropy, enthalpy, and principal component analysis (PCA).

The data described here are related to the article entitled “Molecular Genetics Characterization and Homology Modeling of the *CHM* Gene Mutation: A study on Its Association with Choroideremia” (Imani et al., 2018) [1].

**Specifications Table**

**Table:** 

Subject area	*Computational Biology*
More specific subject area	*Molecular dynamics simulation of the hotspot mutations in choroideremia*
Type of data	*Figure, diagram, table*
How data was acquired	*X-ray crystallographic structure of the Rep-1 protein in complex with 2 monoprenylated Rab 7 protein (PDB code:*1vgo*) was retrieved from RCSB protein data bank. The 3D structure of CHM was generated using MODELLER 9.17 software*[2]*and X-ray crystallographic structure of*1vg0*as a template.*
Data format	*Analyzed*
Experimental factors	*The protein simulations were immersed in SPC water molecules. All covalent bonds to hydrogen atoms were constrained using the SHAKE algorithm. Simulations were conceded using the particle-Mesh Ewald algorithm. The PME method was applied for calculation of the Long-range electrostatics interactions.*
Experimental features	*Homology modeling of primary sequence of Rep-1 protein*
Data source location	*Luzhou, Sichuan, CN*
Data accessibility	*Data is provided within this article*

**Value of the data**

• Computer-based tools allow the identification of conserved domains and/or function of hot-spot mutated systems.
• Data of residue interaction networks (RIN) can show the differentiation of the various nteraction maps of hotspot mutant-type residue sources under the RIN models.
• Comparative principal component analysis (PCA) modeling analysis in predicted hotspot mutant variants can identify major dynamical differences of CHM protein regions and make appropriate predictions.
• Addressing the question of how the hotspot mutations can contribute to the total dynamic free energy profile in a simulation in mutant conformations of the CHM complex; we assessed the free energy landscape (FEL), entropy and enthalpy analysis, which helps to visualize the stability of mutant conformations for a protein.

## 1 Data

**Fig. 1:**
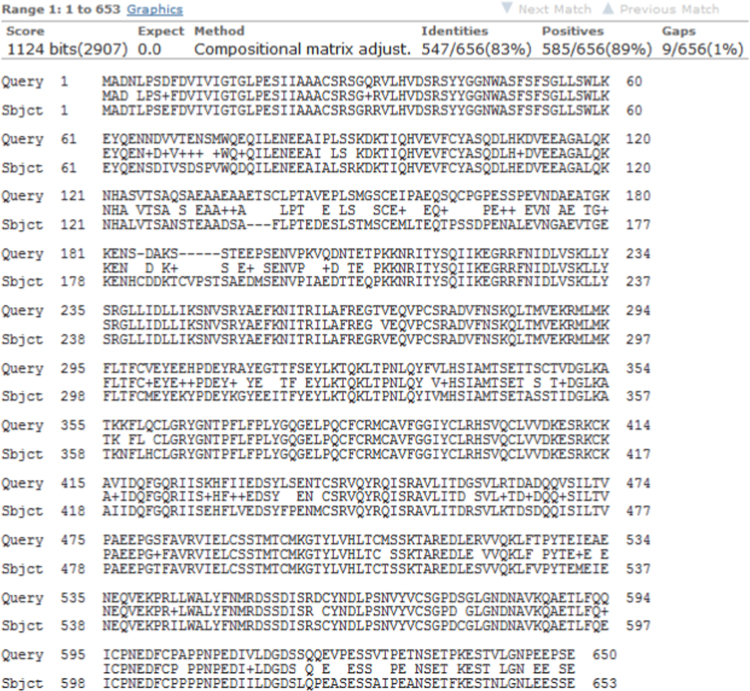
Alignment between template file (1vg0.pdb) and model using EBI server. Query: the sequence investigated, subject: the sequence of template.

**Fig. 2:**
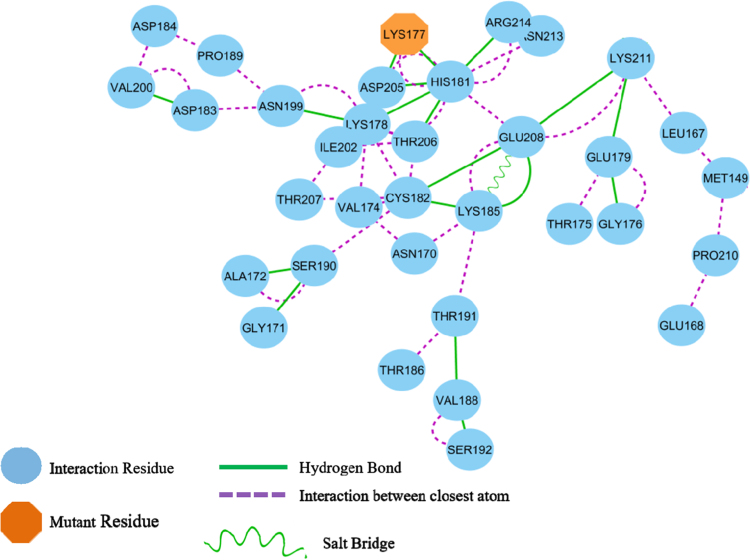
The residue interaction network of the active functional extracellular domain residues of CHM position in the E177K hotspot mutant form.

**Fig. 3:**
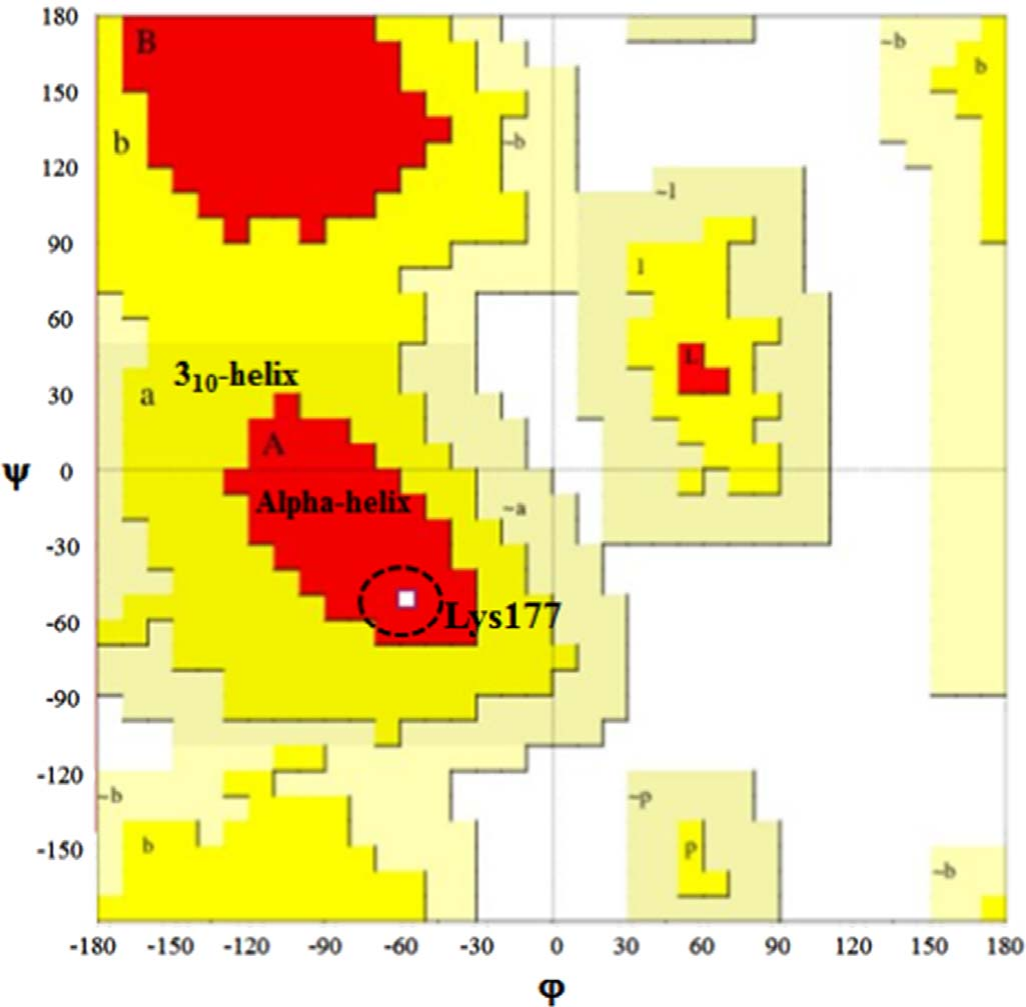
Ramachandran diagram for CHM protein variants in the E177K hotspot mutant model during 40 ns ND simulations. Ramachandran plots show the phi (*ϕ*)-psi (*ψ*) torsion angles for the related residue number 177 of CHM in this structure. Lys residue is shown as square (□) and is restricted to the alpha helix region of plots.

**Fig. 4:**
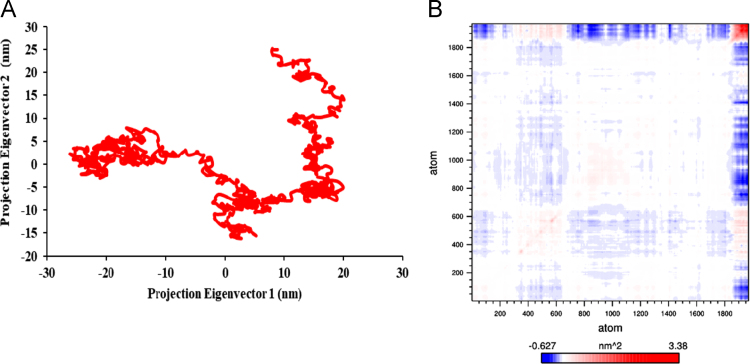
Dynamical effects of E177K hotspot mutation on CHM. PCA scatter plots along the pair of first and second two principal components, PC1 and PC2 for E177K mutant model (A). Cross correlation matrix C-alpha atoms graph and plot in during 40 ns simulation for mutant type (B).

**Fig. 5:**
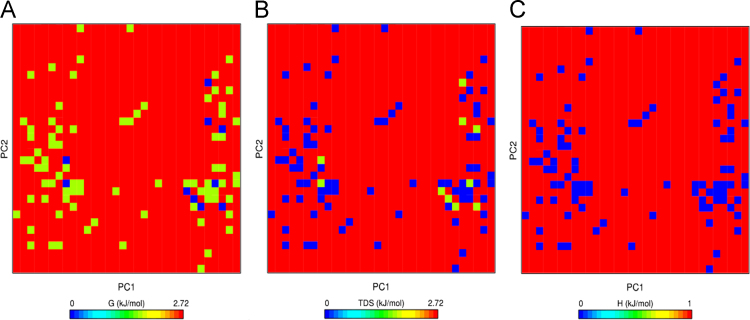
Projections of FEL (A), entropy (B) and enthalpy (C) of E177K hotspot mutant. The dark blue indicates the lowest energy configuration and green shows the middle energy configuration.

The raw data for the human Rep-1 [1] (UniProtKB: P24386.3) are given in protein database. Fig. 1 shows the primary sequence of alignment between templates file (1vg0.pdb), which was obtained from EBI server [3]. This primary sequence compares closely to the GenBank sequence with 83% sequence identity. The relationship between the key residues of E177K hotspot mutant by generating RINs in during the 40 ns of MD running is showed in Fig. 2. Comparison between the predicted key residues of the mutated Lys in the 177th position revealed that the mutant residue has two sites of hydrogen bonding with residues Asp205 and His181 (Fig. 2). Also, the mutant form of CHM was noticeably stabilized through closest atom interactions of Asp205 and His181 (Fig. 2). The Ramachandran plot of E177K hotspot mutant type is provided in Table 1 and Fig. 3. Fig. 4A shows a scatter plot constructed E177K hotspot mutant model in both types' eigenvectors, PC1 and PC2; whereas a graph of the correlated and anti-correlated motions in the mutant system is displayed in Fig. 4B. Clearly, the E177K mutant structure shows significantly higher atomic density distribution and a larger phase space. Clearly, the E177K mutant structure shows significantly higher atomic density distribution and a larger phase space. A model of free energy landscape is summarized in Fig. 5.

**Table 1:** The Ramachandran plot assessment of the E177K hotspot mutant model.

	**Favored region (%)**	**Favored a.a. number**	**Allowed region (%)**	**Allowed a.a. number**	**Outlier region (%)**	**Outlier a.a. number**
**E177K Mutant**	74.9	188	18.7	47	6.4	16

Abbreviations: a.a: amino acids; E177K, Glutamic acid substitution conserved Lysine at codon 177.

## 2 Experimental design, materials and methods

### 2.1 Structural modeling of the hotspot mutant-type in residue interaction networks

To produce the data, a structural model of CHM was generated using MODELLER [2], then the average structure was derived from the 40 ns trajectory of the system; the E177K hotspot mutant was used to construct the RINs in 2D graphs using RING 2.0 web server [4]. The model was viewed using PyMol software [5]. The influence of important hotspot nonsense mutations on the creation of CHM disease was analyzed using chimera software [6]. We tested the relationship between the key residues of E177K hotspot mutant by generating RINs in during the 40 ns of MD running.

The protein simulations were immersed in Simple Point Charge (SPC) water molecules [7] in a cubic box that consist of counter Na^+^ and Cl^−^ ions. All covalent bonds to hydrogen atoms were constrained using the SHAKE algorithm. Simulations were conceded using the particle-Mesh Ewald algorithm [8]. The Ramachandran plot of E177K hotspot mutant type is provided in Table 1 and Fig. 3. Of the model residues, 18.7% of the residues of the E177K hotspot mutant model were placed in the allowed regions. Furthermore, the residues located in the favored zone were also increased to 74.9% during the MD simulation.

As shown in Fig. 3, after Lys177 substitution, the new residue is located in the alpha helix part of the plot.

### 2.2 Dynamical cross-correlation coefficients of hotspot mutant model

To produce the data, two principal motions were considered to evaluate the general fluctuations; the first principal motion (PC1) corresponding to the scissoring motion between the interacting residues, and another one (PC2) indicating a twisting motion. The 40 ns molecular dynamics (MD) simulation of hotspot mutation conformational diversity on the functional active domain of CHM was performed using the GROMACS MD package version 5.1.3 [9]. Our MD protocol has been previously described in literature [1].

### 2.3 Data from a model of free energy landscape

To calculate electrostatic interactions, each system was energy-minimized using the steepest descent algorithm for 10,000 steps with a maximum force smaller than that of 1000.0 kJ mol^−1^ nm^−2^. The system was equilibrated using position-restrained [10] MD with 100 ps of isochoric-isothermal (NVT) equilibration and isothermal-isobaric (NPT) ensemble under the condition of position restraint for heavy atoms, with 100 ps with gradual heating from 300 K [11]. All bonds lengths were constrained using LINCS (Linear Constraint Solver) algorithm [11]. Also, Berendsen thermostat [12] and Parrinello-Rahman algorithm [13] were utilized to control temperature and pressure, respectively. The Particles Mesh Ewald (PME) method [8] was applied for calculation of the Long-range electrostatics interactions. Finally, analyses scripts were visualized and derived from VMD repositories [14]. To identify whether the mutation of E177K can modify the total free energy profile of CHM protein, the interaction energy, function of the enthalpy, and entropy of protein in mutant conformations of CHM protein were analyzed out using GROMACS package software based on the PCA data. These data are summarized in the Fig. 5A, where the energy distribution is a centralized form, indicating the overall conformational stability of the system. Comparatively, the lowest free energy for the system was found to be 0.32 Kcal/mol (Fig. 5A). These results were confirmed through entropy and enthalpy graphs (Fig. 5B and C, respectively).
