# Supplementary material for: In silico data analyses of the hotspot mutations of CHM gene in choroideremia disease
Source: Data Brief. 2018 Apr 12;18:1217–23. doi: 10.1016/j.dib.2018.04.023 (PMC5997011; doi:10.1016/j.dib.2018.04.023)
Supplement: Supplementary file 1 — Supplementary material [file mmc1.docx]

Dear Editor;

The authors declare no conflict of interest on revise our paper entitled, "**In silico data analyses of the hotspot mutations of *CHM* gene in choroideremia disease**.

Junjiang Fu, Ph.D

Professor and director

Key Laboratory of Epigenetics and Oncology,

The Research Center for Preclinical Medicine,

Southwest Medical University,

Luzhou, Sichuan, China;

E-Mail: [fujunjiang@hotmail.com](mailto:fujunjiang@hotmail.com).
